# Behavioural responses of the hagfish *Eptatretus stoutii* to nutrient and noxious stimuli

**DOI:** 10.1038/s41598-019-49863-x

**Published:** 2019-09-16

**Authors:** Chris N. Glover, Dustin Newton, Jasmin Bajwa, Greg G. Goss, Trevor J. Hamilton

**Affiliations:** 1 Athabasca River Basin Research Institute and Faculty of Science and Technology, Athabasca University, Athabasca, Alberta Canada; 2 Department of Biological Sciences, University of Alberta, Edmonton, Alberta Canada; 3 Bamfield Marine Sciences Centre, Bamfield, British Columbia, Canada; 4 Department of Psychology, MacEwan University, Edmonton, Alberta Canada; 5 Neuroscience and Mental Health Institute, University of Alberta, Edmonton, Alberta Canada

**Keywords:** Olfactory system, Marine biology

## Abstract

The suitability of a traditional testing paradigm (e.g. choice chamber) for assessing chemosensory behaviour in the Pacific hagfish, *Eptatretus stoutii*, was examined. Actively-swimming hagfish, tested at night, showed no preference for any region of a T-maze in the absence of a stimulus, but in the presence of an olfactory food cue, spent significantly more time in the zone where the cue was placed. Conversely, hagfish avoided spending time in the zone the fish anaesthetic 3-amino benzoic acid ethylester (MS-222) was placed, and demonstrated significantly more reversal responses in which the fish moved its body backwards. These data suggest that hagfish are an amenable model species for laboratory testing of behaviour.

## Introduction

Hagfish display feeding behaviours that are considered unusual relative to other vertebrates^1^. These evolutionarily ancient benthic chordates are opportunistic scavengers that bore into, and immerse themselves within, decaying carcasses, subsequently eating their way from the inside out. Because of this feeding mode they undergo long periods of fasting between meals^2^, and within the body cavity, maximisze nutrient uptake by using their integument to absorb dissolved nutrients arising from decay^1^. However, hagfish also exhibit other feeding behaviours more characteristic of vertebrates. For example, the use of video at baited traps has shown that hagfish can actively prey on small fish^3^. This observation has been corroborated by gut content analyses, which also indicate predation extends to invertebrates (see^1^). Regardless of feeding mode, the detection and localisation of sea-floor carrion and/or prey items by hagfish must require well-adapted chemosensory behaviours. Chemosensation is likely to be particularly important for hagfish given their habitation of deep waters with limited light, and their poor visual capabilities^4^. Although there is some penetration of blue light to the depths that hagfish inhabit, the lack of a lens or iris in the hagfish eye means that they lack the capacity for image forming.

Currently there is only a rudimentary understanding of chemosensory systems in hagfish. However, evidence to date indicates that it is indeed an important factor associated with feeding. For example, field studies show that hagfish are invariably one of the first, and subsequently the most numerous, of species to attend baited underwater camera set-ups. In one study the majority of hagfish appeared at a carcass within 15 minutes, half the time noted for the majority of the next fish species to arrive^5^. This suggests a strong capacity for detection of chemical signals associated with decay, a finding supported by observational studies in laboratory settings that show positive chemotactic behavioural responses in response to decay-related chemoattractants^2^. These observations are also consistent with knowledge of the neurobiology of hagfish chemosensory systems, which likely consist of olfaction, and a specialised and unique system of sensory organs called Schreiner organs. While hagfish olfaction is known to be sensitive to typical chemoattractant molecules (i.e., amino acids)^6^; the function of the Schreiner organs is unknown. However, their structure and innervation, and their wide distribution over the body surface of hagfish, indicates that they are likely to perform an important role in chemosensation^7^.

To date, studies on hagfish chemosensory behaviour have been restricted to largely subjective, observational studies. While such studies offer useful insight into hagfish behaviours they do not necessarily facilitate the objective testing of specific hypotheses, under controlled laboratory conditions. The use of behavioural testing paradigms, in association with objective and quantifiable endpoints, is critical for developing an improved understanding of hagfish ecology and physiology. In the current study, the viability of a traditional behavioural test apparatus (the T-maze) for measuring hagfish behaviour was assessed. Pacific hagfish (*Eptatretus stoutii*) responses to either a food cue (blended herring) or a noxious stimulus (the fish anaesthetic 3-aminobenzoic acid ethyl ester methanesulfonate (MS-222), also known as tricaine methanesulfonate (TMS) or tricaine), were examined, in order to determine the ability of hagfish to respond to chemosensory stimuli. This anaesthetic is the most widely used agent for anaesthesia and euthanasia in hagfish biology. Aversive reactions to MS-222 have been reported in some teleosts, and this chemical has been previously used as a model aversive substance in the study of fish behaviour^8^. However, this aversive response is not universal among fish^9^. Anecdotally, authors of the current work have previously noted a strong negative behavioural response upon immersion of hagfish in MS-222, suggesting this chemical is an appropriate model noxious stimulus for assessing behavioural avoidance. Our study sought to test the hypothesis that hagfishes are amenable to behavioural testing, and that they would exhibit a positive chemotaxis towards food cues, and a negative chemotaxis towards a noxious chemical when assessed in a traditional behavioural choice chamber apparatus.

## Materials and Methods

### Animals

Pacific hagfish (*Eptatretus stoutii*) were captured at a depth of ~50 m via a trap baited with hake (*Merluccius productus*), placed in Barkley Sound, Vancouver Island, Canada. Thereafter, hagfish were held in 100-L tanks receiving flow-through seawater at 12 °C. These outdoor tanks were subject to natural light conditions, but remained covered with heavy-duty plastic lids to ensure low light conditions within the tank. Animals were held unfed until experimentation (approximately two weeks). Six hagfish (average length 51.8 ± 3.0 cm) were used in this study. Behavioural studies were all performed at night (10 PM–3 AM), consistent with the nocturnal activity patterns of this species^4^. All experiments were performed in accordance with the relevant guidelines and regulations under a University of Alberta Animal Use Protocol (AUP 00001156), and by approval from the BMSC Animal Care and Use Committee (RS-17-03).

### Behavioural assay

**Figure 1:**
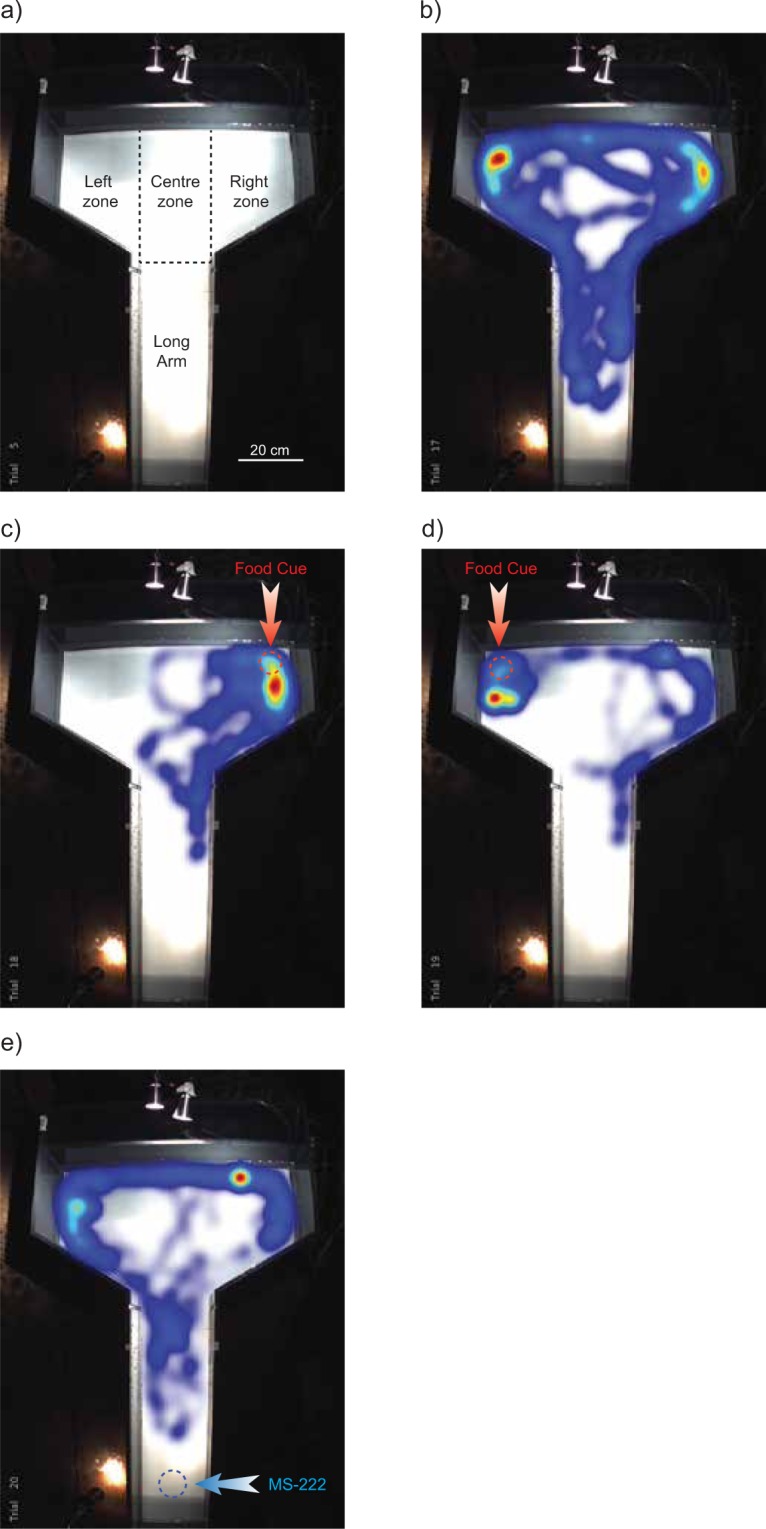
Images of modified T-maze arena and individual hagfish zone preference during each trial. (**a**) Image of modified T-maze arena with an overlay of the zones used in the motion-tracking software system to quantify zone preference. (**b**) Heatmap (visual representation of location of hagfish over the trial) of one hagfish in Trial 1 with no cues present. (**c**) Heatmap of the movement of one hagfish after the presentation of the food cue in Trial 2. The arrow and dotted circle represent the location of the placement of the food cue. (**d**) Heatmap of the movement of one hagfish after the placement of the food cue in the opposite zone in Trial 3. The arrow and dotted circle again represents the location of the placement of the food cue. (**e**) Heatmap of the movement of one hagfish after the placement of MS-222 in the long arm of the arena (Trial 4). The arrow and dotted circle again represents the location of the placement of the MS-222.

A modified T-maze arena was used for all behavioural trials (72 cm wide at the top, 20 cm wide along the trunk, 106.5 cm long, see Fig. 1a). The arena was constructed of 2 cm thick grey plastic, with white corrugated plastic on the bottom of the arena to maximize contrast for the motion-tracking software system (Ethovision XT, v.10; Noldus Information Technology, Leesburg, VA, USA), and was freshly filled with water from the hagfish holding tanks for every hagfish tested. A constant low level of incandescent lighting was used to allow the motion-tracking software to decipher between the dark hagfish and the light background. Swimming hagfish were transferred from holding tanks to the maze, by placing a transfer container (white pail) in the holding tank and allowing the fish to swim into the container, while moving the container in the direction of hagfish movement. Hagfish usually maintained their swimming behaviour while the pail was taken into the testing room where they were gently placed into the center of the arena. If the hagfish stopped swimming and formed a natural resting coiled position either in the pail or in the arena then the hagfish was placed back into the holding tank and another hagfish was used. Due to the challenge of maintaining hagfish in a swimming state, trials 1–4 were performed in succession on each hagfish. Trial 1 assessed hagfish movement in the absence of any cue. This trial began five minutes after the hagfish was placed into the arena and involved recording hagfish movement for five minutes (Fig. 1b). Next, when the hagfish swam into the long arm of the arena, a food cue was delivered into the left or right zone (chosen at random using a Microsoft Excel randomize tool) and the behavioural response to this cue was recorded over the subsequent 5 minutes (Trial 2; Fig. 1c). Food cues were created prior to testing by blending Pacific herring (*Clupea pallasii*) into a slurry, centrifuging and collecting the supernatant. This cue was delivered to the maze via a pipette in 1-mL doses on the surface of the water. Five minutes after the initial food cue, a second food cue was delivered into the opposite zone (i.e. if the initial food cue was placed in the left zone, then the second cue was placed in the right zone, and vice versa). This second food cue trial constituted Trial 3, which again involved monitoring of behavior for a five minute period (Fig. 1d). The fourth and final trial involved the placement of the noxious cue of MS-222 (10 g in 200 mL of seawater, neutralized with NaOH). This cue was added at the base of the long arm of the arena while the hagfish was in the open area of the arena (left, right, or center zones; Fig. 1e). Again, this trial lasted for five minutes. Full water changes were performed after each set of trials per hagfish. Average velocity (cm/s), and time in zones (s) were calculated for each minute of the five minute trial and were quantified in Ethovision XT similar to previous studies^10,11^.

### Statistical analysis

**Figure 2:**
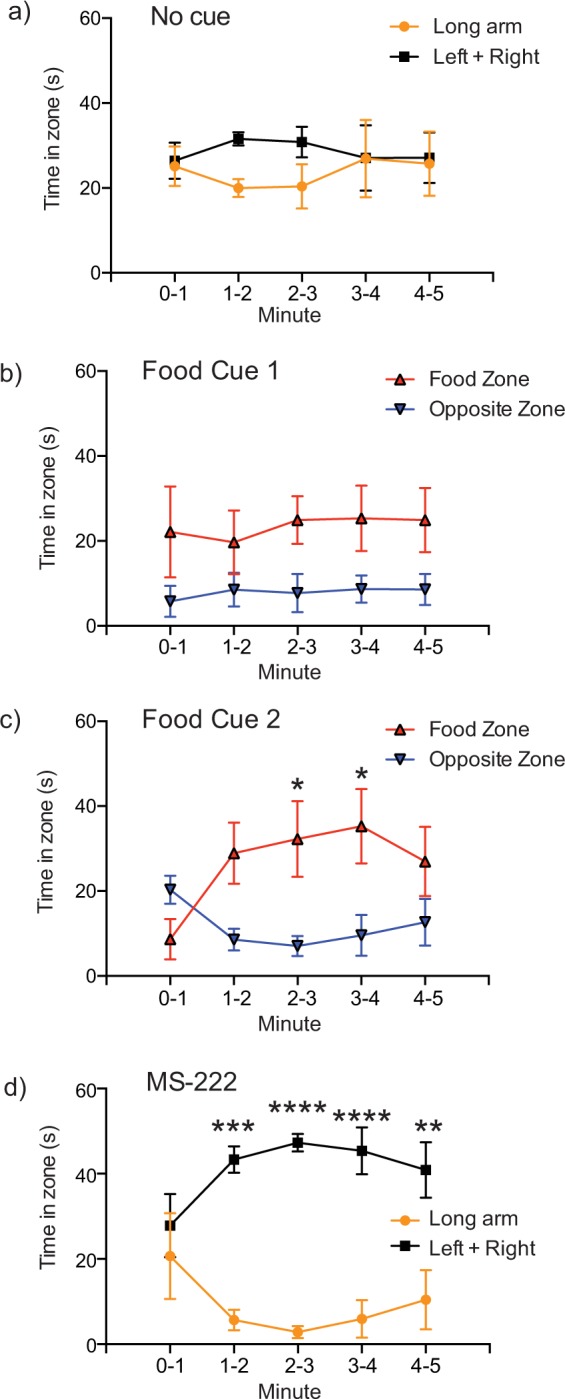
Location preference of hagfish in experimental conditions measured every minute of the trial. (**a**) With no stimulus administered the hagfish had no preference for the equally sized left + right zones or the long arm of the arena (F(1.10) = 0.7649, P = 0.4023). (**b**) The addition of the food cue caused a significantly higher time spent in the food zone compared to the equally sized opposite zone (F(1,10) = 5.237, P = 0.0451). (**c**) Subsequent placement of the food cue into the previously opposite zone caused a significantly higher time spent in the location where the second food cue was administered (F(1,10) = 11.63, P = 0.0067). Post hoc testing revealed a significant location preference at 2–3 and 3–4 minutes (*P < 0.05). (**d**) The placement of MS-222 into the long arm of the arena resulted in a significantly higher time spent in the left + right zones relative to the equally sized long arm zone (F(1,8) = 63.28, P < 0.0001). Post hoc testing revealed a significant location preference at 1–2 mins (*** P < 0.001), 2–3 mins and 3–4 mins (****P < 0.0001), and 4–5 mins (**P < 0.01).

**Figure 3:**
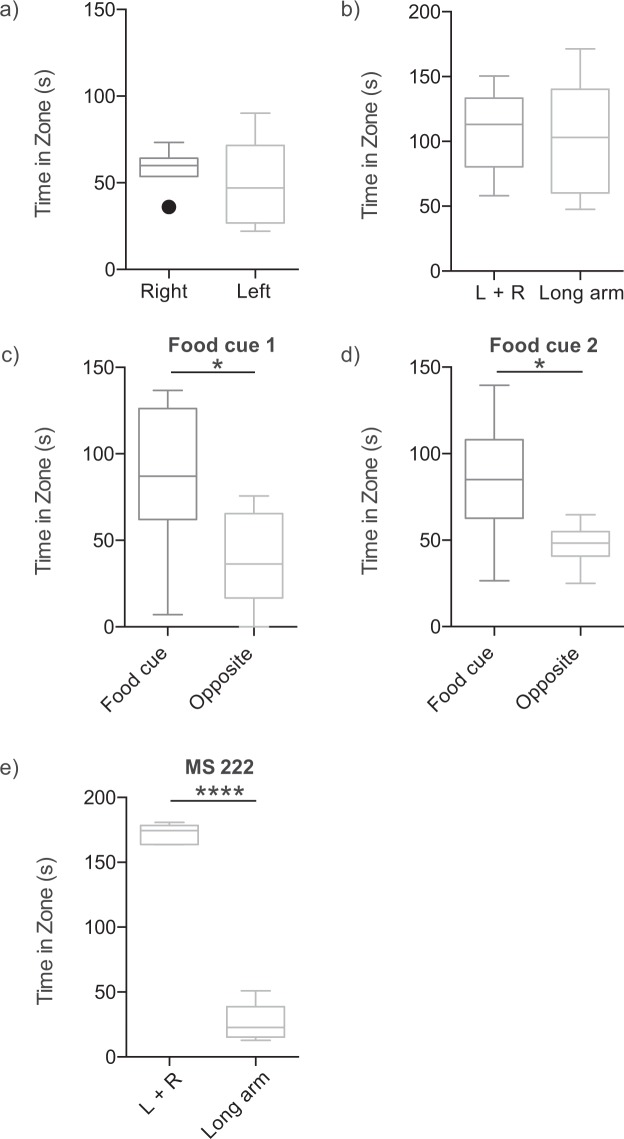
Location preference of hagfish in experimental conditions over the first four minutes of the trial. (**a**) With no stimuli in the arena the hagfish did not display a significant difference in time spent in the left compared to the right zone (P = 0.4740). (**b**) With no stimuli in the arena the hagfish did not display a significant difference in time spent in the equally sized or left + right zones compared to the long arm zone (P = 0.8284). (**c**) After the placement of the food cue the hagfish spent more time in the zone where the food cue was administered compared to the opposite zone (*P = 0.0172). (**d**) Subsequent placement of the food cue into the previously opposite zone resulted in a significant preference in the location where the second cue was presented (*P = 0.0480). (**e**) MS-222 placed into the long arm resulted in a significant preference for the left + right zones compared to the equally sized long arm zone (****P < 0.0001). All graphs are box and whisker plots.

Two-way ANOVAs were used for minute-by-minute analyses with Sidak’s multiple comparison post hoc test, where trial time and apparatus zone were the independent variables, and time spent in each zone (zone location) by the hagfish was the dependent variable (Fig. 2). We also used unpaired t-tests to compare specific zones of interest in each trial, and one-way ANOVAs with Tukey’s HSD multiple comparison post hoc test to compare velocity data across Trials 1–4 (Fig. 3). We excluded the last minute of the trial in the unpaired t-tests and the one-way ANOVA analysis because the food cues may have lost effectiveness in the last minute according to our minute-by-minute analyses. To analyse movement throughout the arena without the presence of any stimulus we combined the left and right zones (720 cm^2^ + 720 cm^2^; “top zone”) as they are equivalent in area to the long arm (1440 cm^2^) which allowed us to examine whether there was an innate preference for the more open “top zone” relative to the longer and thinner “long arm” in Trial 1. The “centre zone” was not included in the sum because the area would then be nonequal. The sum of the left and right zones was also used when MS-222 was placed into the “long arm”. To examine whether hagfish were attracted to food cues we compared the zone that the stimulus was administered in to the identically sized opposite zone that contained no cues. Significantly more time in the food zone would indicate a preference for the food cue. To quantify “avoidance behaviour” potentially due to the MS-222 we also examined “reversal responses” by manually scoring the instances that the anterior of the fish moved in the opposite direction of the swim direction (best described as an erratic backwards jerking motion not due to contact with the wall of the arena). An alpha-level of 0.05 and 95% confidence intervals were used for assessing statistical significance in all tests. All graphs are box and whisker plots with the Tukey’s method for plotting whiskers and outliers. Data were analysed using GraphPad PRISM v6.0 (San Diego, CA, USA). For one hagfish in the MS-222 trial, the animal ceased swimming, assumed a natural rested coiled position in the arena, and did not resume activity. Data from this hagfish were excluded from analysis for time in zones and velocity.

## Results

When placed in a modified T-maze, and in the absence of any stimulus, actively swimming hagfish explored the apparatus (Supp. Video 1, Fig. 1b), without displaying statistical preference for either the left + right (top zone), or long arm when we quantified each minute (Fig. 2a; F (1,10) = 0.7649, P = 0.4023). When we assessed the first 4 minutes of the trials combined, again we found no difference in the left or right zones (Fig. 3a,b; right zone: 58.3 ± 5.0 s, left zone: 50.0 ± 10.4 s, n = 6, P = 0.474) or left + right versus long arm zones (Fig. 3b: left + right zone: 108.4 ± 13.6; long arm zone: 103.3 ± 18.4, n = 6, P = 0.8284).

Following the introduction of a food cue into either the left or right zone (Supp. Video 2, Fig. 1c), a minute-by minute analysis (i.e. two-way ANOVA with time and zone location as the two-factors) found that hagfish preferentially spend more time in the zone in which the food cue was placed, relative to the opposing zone where food cue was not placed (F (1,10) = 5.237, P = 0.0451) (Fig. 2b). In a second food cue trial where the food cue was placed in the opposite zone to food cue trial 1 (Supp. Video 3, Fig. 1d), the hagfish again demonstrated a preference for the food cue zone (F (1,10) = 11.63, P = 0.0067) (Fig. 2c). When we analysed these data using total pooled time over the first 4 minutes of the trials the same outcome was noted; in food cue trial 1, hagfish spent more time in the zone in which the food cue was placed (86.8 ± 18.4 s), relative to the opposing zone where food cue was not placed (38.7 ± 11.3 s; P = 0.0172, n = 6) (Fig. 3c). In the second food cue trial the hagfish again demonstrated a preference for the food cue zone (food cue zone: 84.7 ± 15.0, opposite zone: 47.3 ± 5.2 s, n = 6, P = 0.048) (Fig. 3d).

In the presence of MS-222, a model noxious stimulus, hagfish displayed avoidance behaviour (Supp. Video 4, Fig. 1e). The placement of MS-222 in the long arm of the T-maze resulted in a significantly greater period of time spent in the left + right zones of the maze compared to the long arm zone (two-way ANOVA; F (1,8) = 63.28, P < 0.0001) (Fig. 2d). When time was pooled across the first 4 minutes of the trials (i.e. analysis via one-way ANOVA), hagfish spent a significantly greater period of time in the left + right zones of the arena (left + right zones: 171.8 ± 3.4 s, long arm zone: 26.2 ± 6.6 s, n = 5, P < 0.0001) (Fig. 3e). Neither the food cue nor MS-222 significantly affected swimming velocity (F(3,19) = 1.762, P = 0.1884, Fig. 4a).

**Figure 4:**
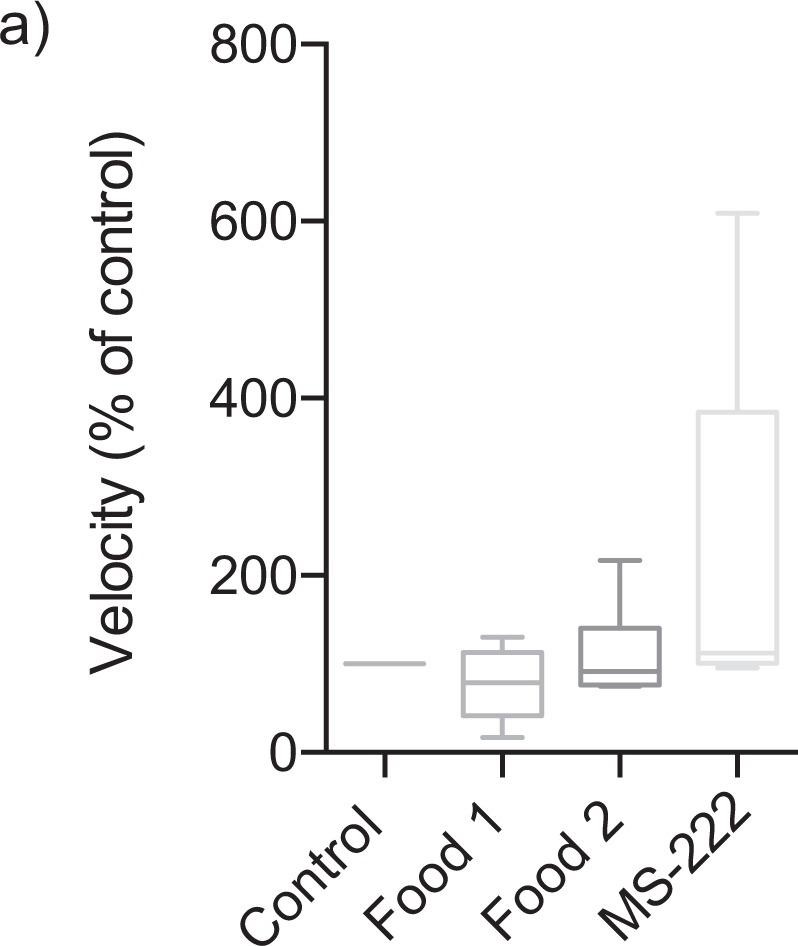
Velocity throughout all trials. (**a**) Velocity in each trial involving cue placement was divided by the velocity in the first trial (control) for each hagfish. There was no significant change in velocity caused by any of the stimuli added to the arena. (F(3,19) = 1.762, P = 0.1884, box and whisker plot).

**Figure 5:**
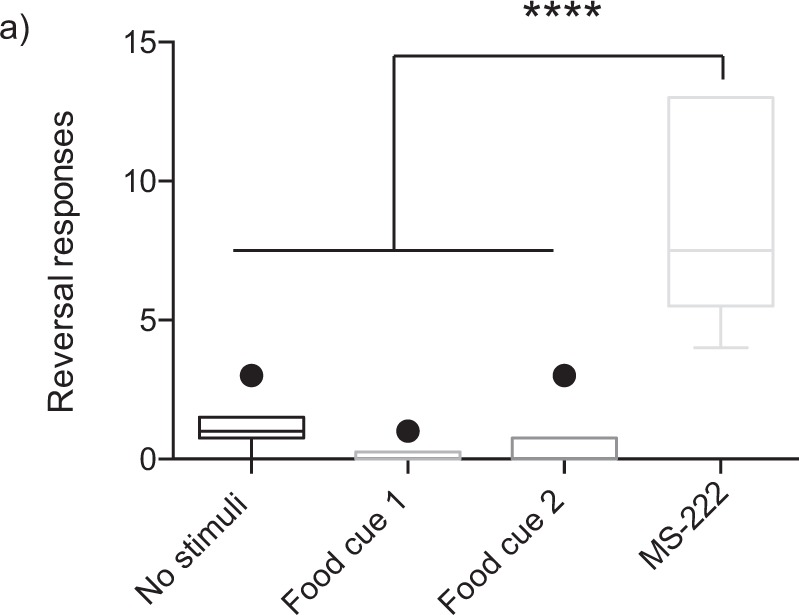
Reversal responses in each trial. (**a**) To quantify potential aversion to the MS-222 we counted the number of ‘reversal responses’ (see Methods for more detail) in each trial. The number of events was significantly higher in the MS-222 trial compared to all other trials. (F(3,20) = 21.78, ****P < 0.0001, box and whisker plot).

To further quantify any potential aversive responses we determined the number of ‘reversal responses’ in each of the full trials. There was a significant difference in reversal responses (Fig. 5a; F(3,20) = 21.78, P < 0.0001) with post hoc analysis showing a significantly greater number of reversals in the MS-222 trial (8.5 ± 1.5) relative to all others (Trial 1: 1.2 ± 0.4, Trial 2: 0.2 ± 0.2, Trial 3: 0.5 ± 0.5).

## Discussion

### Hagfish responses to food cues

As hypothesized, hagfish responded positively to the presence of a food cue, spending a significantly longer time in the zone of a T-maze where the food cue was placed, than in opposite zone of the test apparatus (Fig. 2). This establishes the suitability of this testing paradigm for examining behavioural responses of hagfish, consistent with findings of teleost fish in T-maze systems where food is used as a behavioural inducement^12^. Importantly, the responses of hagfish to food cues correspond with field studies that note the rapid appearance of hagfish at baited camera locations^5^. This behaviour is likely driven by chemosensation, given the long distances over which this response may occur, and the relatively well-developed chemosensory neurobiology of hagfish^7^.

Our data are also consistent with previous laboratory studies of hagfish responses to food cues. Tamburri and Barry^2^ observed that hagfish responded in a dose-dependent manner to cues placed directly in front of the nasal cavity. Quantification of dissolved free amino acid concentrations in the supplied cue of that study, showed that the threshold of the behavioural response in hagfish was 6.5 µM. To place this in an environmental context, concentrations of amino acids in seawater are generally in the nM range^13^. While higher amino acid concentrations would be anticipated at a decaying source, the dilution profile from source to receptor is unknown, and the distances over which hagfish may be able to detect sea-floor carcasses remains unstudied. The most notable aspect of the 6.5 µM behavioural response threshold is that this value is significantly lower than the 100 µM concentration required to induce electrical responses in the olfactory nerve of hagfish^6^. This suggests that olfaction alone is unlikely to be the driver of the chemosensory response. The goal of the current study was to validate the use of behavioural choice paradigms to study hagfish behaviour, and therefore we did not attempt to characterise the sensitivity of the response, nor identify the nature of the chemoattractant. However, our data suggest that the T-maze is an appropriate paradigm for future studies seeking to assess the nature and dose-dependency of hagfish responses to specific putative chemoattractants.

### Hagfish responses to MS-222

Actively swimming hagfish spent significantly more time in zones that did not contain the fish anaesthetic (Figs 2d, 3e), consistent with our hypothesis. However, the zones that did not contain MS-222, previously contained the food cues, so the most relevant finding is the reversal responses in the MS-222 trials (Fig. 5a). The significant increase in observed reversal responses in the presence of the putative noxious stimulus, relative to nutrient stimuli and the absence of chemical cues, is strong evidence that hagfish sense and enact avoidance behaviours in response to the fish anaesthetic. This finding is consistent with the use of MS-222 as a model aversive chemical in teleost fish^8^. Given its lipophilicity, MS-222 will rapidly traverse the gill and skin epithelia of hagfish, entering the circulation. As it is distributed around the body, anaesthesia is induced by blockade of voltage-gated sodium channels in nervous tissue^14^. However, it is unlikely that the anaesthetic mode of action is also the mechanism responsible for the aversive response. Owing to distinct behavioural responses to MS-222 between species with presumably similar neurophysiology, it has been suggested that the aversive effects of MS-222 are instead a consequence of its physical properties, and are mediated by interactions with chemosensory moieties^15^.

The outcomes of behavioural experiments with MS-222 not only show the capacity of hagfish to display aversive behaviour towards a noxious cue, but may also have implications for animal welfare. Aversion responses of MS-222 are seen in a number of teleost fish, most notably zebrafish^9^. Such behaviours are indicative of stress, and on this basis the use of MS-222 as an agent for anaesthesia and euthanasia in zebrafish has been questioned^9,15^. Although the velocity of the hagfish was not significantly increased by MS-222, future studies should consider a larger and circular testing arena as ours may have been too small to detect velocity changes. Given hagfish also show aversive behaviour to MS-222, then anaesthesia and euthanasia with this chemical in this species is likely to be stressful, and thus contrary to animal welfare best practice. Indeed, previous work has shown that exposure of hagfish to an anaesthetic mixture of MS-222 and benzocaine stimulates physiological markers of stress, with a more than 100-fold increase in plasma norepinephrine relative to rested controls^16^. Future studies should investigate the efficacy of MS-222 in hagfish, and the effectiveness of alternate mechanisms of anaesthesia/euthanasia such as clove oil-based formulations^17^, to ensure suffering of the animal is minimised.

### Summary and perspectives

Confirming our initial hypothesis, the current study showed that the behaviour of hagfish is amenable to investigation via traditional laboratory testing paradigms, such as the T-maze. The methods used here could be easily used to further investigate key questions regarding feeding behaviour in hagfish (i.e. the sensory modalities involved; sensitivity to, and nature of, the chemoattractants involved), and other aspects of hagfish chemosensory and cognitive biology. Given the value of hagfish as an evolutionary model species, there is also significant scope for using these paradigms to understand the evolution of behaviours and neurological systems in vertebrates. Hagfish are the most ancient of extant vertebrates, thought to have diverged from the main line of vertebrate evolution as many as 560 million years ago. While many of their features are considered to be degenerate relative to a shared common vertebrate ancestor^18^, they nevertheless represent a valuable tool for the study of evolution of vertebrate traits. Consequently, the study of hagfish behaviour under controlled laboratory conditions would represent an important tool aiding in the understanding the evolution of a range of behavioural phenomena, ranging from chemosensation to cognition.

## Supplementary information

Supp. video 1

Supp. video 2

Supp. video 2

Supp. video 2
